# Single Ascending Dose Safety and Pharmacokinetics of CDRI-97/78: First-in-Human Study of a Novel Antimalarial Drug

**DOI:** 10.1155/2014/372521

**Published:** 2014-03-27

**Authors:** N. Shafiq, S. Rajagopalan, H. N. Kushwaha, N. Mittal, N. Chandurkar, A. Bhalla, S. Kaur, P. Pandhi, G. D. Puri, S. Achuthan, A. Pareek, S. K. Singh, J. S. Srivastava, S. P. S. Gaur, S. Malhotra

**Affiliations:** ^1^Department of Pharmacology, PGIMER, Chandigarh, India; ^2^Pharmacokinetics and Metabolism Division, Central Drug Research Institute, Lucknow, India; ^3^IPCA Laboratories Ltd., Mumbai, Maharashtra, India; ^4^Department of Internal Medicine, PGIMER, Chandigarh, India; ^5^Department of Anaesthesia Incharge, Critical Care, PGIMER, Chandigarh, India; ^6^Clinical and Experimental Medicine, Central Drug research Institute, Lucknow, India

## Abstract

*Background*. CDRI 97/78 has shown efficacy in animal models of *falciparum* malaria. The present study is the first in-human phase I trial in healthy volunteers. *Methods*. The study was conducted in 50 healthy volunteers in a single, ascending dose, randomized, placebo-controlled, double blind design. The dose ranges evaluated were from 80 mg to 700 mg. Volunteers were assessed for clinical, biochemical, haematological, radiographic, and electrocardiographic parameters for any adverse events in an in-house facility. After evaluation of safety study results, another cohort of 16 participants were administered a single oral dose of 200 mg of the drug and a detailed pharmacokinetic analysis was undertaken. *Results*. The compound was found to be well tolerated. MTD was not reached. The few adverse events noted were of grade 2 severity, not requiring intervention and not showing any dose response relationship. The laboratory and electrocardiographic parameters showed statistically significant differences, but all were within the predefined normal range. These parameters were not associated with symptoms/signs and hence regarded as clinically irrelevant. Mean values of *T*_1/2_, MRT, and AUC_0−*∞*_ of the active metabolite 97/63 were 11.85 ± 1.94 h, 13.77 ± 2.05 h, and 878.74 ± 133.15 ng*·*h/mL, respectively *Conclusion*. The novel 1,2,4 trioxane CDRI 97/78 is safe and will be an asset in malarial therapy if results are replicated in multiple dose studies and benefit is shown in confirmatory trials.

## 1. Introduction

The global burden of malaria can be estimated from the fact that nearly 107 countries and territories are at risk of malaria transmission and the annual worldwide cases of acute illness due to malaria are estimated to be around 300–500 million [1]. In the Southeastern Asian Region of the World Health Organization (WHO), 1.2 billion are exposed to the risk of malaria, most of whom live in India [2]. In many regions, including India, multidrug resistant* P. falciparum* is one of the important causative strains of malaria.* Falciparum* malaria, which was traditionally effectively treated by chloroquine, now commonly shows resistance to the drug [3]. Till date progress in malaria therapy has been made using the endoperoxide sesquiterpene artemisinin and its derivatives like arteether, artemether, artesunate, and so forth. The World Health Organization (WHO) recommended artemisinin based therapies as first line of therapy for* falciparum* malaria [3, 4].

**Figure 1:**
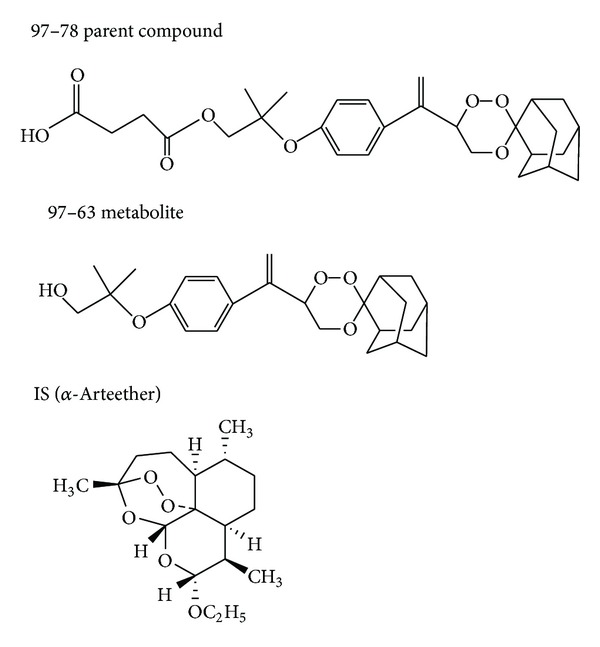
Chemical structure of parent compound 97/78,* in vivo* active metabolites 97/63, and IS (*α*-arteether).

A novel trioxane 97/78, developed by Central Drug Research Institute (CDRI), India, has shown promising antimalarial activity and is currently in clinical trials phase I. 97/78 contains 1,2,4-trioxane nucleus similar to artemisinin responsible for their pharmacological activity [5–9] (Figure 1). During the last 10 years, antimalarials based on inhibition of plasmodial phospholipid metabolism were identified and developed by CDRI groups [5–8, 10–17]. Many compounds have shown high efficacy against multidrug resistant* Plasmodium falciparum* strains [6, 12, 13, 15, 17]. But 97/78 emerged as lead compound for excellent pharmacological activity in both* in vitro* and* in vivo* malarial models. After successful completion of regulatory preclinical studies compound is currently in phase I clinical trials [18, 19].

Potent antimalarial activity of these compounds may be due to its capacity (i) to mimic the choline structure which blocks phosphatidylcholine biosynthesis and (ii) to interact with hemozoin, the malarial hemoglobin-degradation product. Compounds potency is also due to its high accumulation inside the infected erythrocytes. This dual mechanism of action of 97/78 should limit the risk of emergence of resistance. The chemical name of the compound is Mono[2methyl2[3(1spiro[tricycle[3.3.1.13,7]decane2,3′-[1, 2,4]trioxan] 6′-yle thenyl) phenoxy] propyl] ester. It has powerful antimalarial activity against chloroquine resistant* P. falciparum in vitro*, chloroquine sensitive* P. berghei *and multidrug resistant* P. yoelii* in mice, and* P. cynomolgi* in rhesus monkeys. It has been developed as a synthetic substitute for antimalarial peroxide artemisinin and its derivatives such as artemether, arteether, and artesunic acid. The CDRI compound 97/78 is effective by oral and intravenous routes and therefore can be used by either route.

Firstly 97/63 was synthesized but, due to its poor bioavailability, it was resynthesized as a hemisuccinate derivative and coded as 97/78. Upon administration of 97/78 it gets converted into its active* in vivo* metabolite 97/63. The concentrations of 97/63 and 97/78 can be measured by validated LC-MS/MS method [19]. The preclinical testing for this compound included pharmacokinetic (PK) studies, animal pharmacology and toxicology, male fertility studies, female reproduction and development toxicity studies, and genotoxicity studies, and the compound was taken up for evaluation in phase I study as it lacked any remarkable toxicity concerns in animal studies.

The aim of this study was to calculate the maximum tolerated dose of the compound in healthy human volunteers along with evaluation of PK profiles of 97/78 and its* in vivo* metabolite 97/63 after oral administrations of 97/78.

## 2. Subjects and Methods

### 2.1. Subjects

The study was approved by the Institute's Ethics Committee. The single ascending dose study and the pharmacokinetic study are registered with Central Trial Registry of India (CTRI/2012/07/002812; CTRI/2012/07/002832). Normal healthy male volunteers in the age group of 18–45 years were invited to join the trial. A detailed informed consent process of the volunteers was undertaken and screening of the volunteers was done after obtaining informed consent. They were recruited through contacts and hospital employees were excluded. The health status of the volunteers was determined by taking medical history, physical examination, routine haematology (haemoglobin, total lymphocyte count, differential count, and platelet count), clinical biochemistry (serum glutamic oxaloacetic transaminase (SGOT), serum glutamic pyruvic transaminase (SGPT), serum bilirubin, serum creatinine, blood urea, serum electrolytes, random blood sugar, total protein, and serum lipid profile), and urine and stool analysis (routine examination and microscopy). The volunteers were asked to report to the study site after 12 hours of fasting for the blood tests. Chest X-ray and 12-lead ECG were also taken. The participants were also screened for human immunodeficiency virus (HIV) and hepatitis B.

The volunteers were excluded if there was a history of having donated blood in the past 3 months, HIV or HBsAg positivity, history of any drug intake in the past 15 days, history of receiving any enzyme-inducing agent in the preceding 15 days, history of participating in any investigational new drug (IND) study in the previous 6 months, history of participating in any non-IND study in the preceding 3 months, history of allergy or hypersensitivity to any medication, history of peptic disorder, chronic smoker >6 cigarettes/beedies per day, alcoholic (history of daily alcohol intake in the last 6 months), history of any drug abuse, tobacco user in any form, history of any chronic disease and taking medication, and history of taking nonallopathic drug in the past 6 months for any illness or history of any major illness in the past (namely, meningitis, hepatitis, tuberculosis, epilepsy, nephritis having received treatment and presently asymptomatic).

The procedure for recruiting participants for single dose PK study was similar to the above. The PK study was undertaken in 16 separate volunteers following the evaluation of safety data for phase I study and evaluation of pharmacodynamics study in animals

### 2.2. Design

This was a single ascending dose, placebo-controlled, randomized, double blind trial. Randomization was carried out by computer generated random number sequence. Randomization was carried out for the drug and the placebo in a 3 : 1 ratio for each dose level. The drugs were dispensed in sealed bottles with the randomization code written on them. Randomization codes were provided in sealed opaque envelopes with the protocol specification that the codes may be opened by the investigator only in case of a serious or severe adverse event.

### 2.3. Starting Dose and Dose Escalation

The starting dose was 80 mg; calculation of starting dose was based on the basis of maximum tolerated dose of 100 mg/kg obtained in rats. This was used to obtain a dose of 96 mg for a 60 kg man. The formulation which was available for a dose below this was of 80 mg and hence taking these factors into consideration a starting dose of 80 mg was chosen. The following dose levels were evaluated: 80, 160, 320, 400, 500, 600, and 700 mg. The starting dose was calculated by allometric scaling from animals. At each dose level, six volunteers received CDRI 97/78 and two received placebo except at 700 mg where only two volunteers were given this dose. At each dose level, volunteers were assessed before proceeding to the next dose. The decision to dose only 2 volunteers was taken, after analysis of data of 600 mg, wherein no remarkable adverse events were noted.

### 2.4. Stopping Rule

The decision to stop the dose escalation was based on the appearance of the dose limiting toxicity (DLT) which was defined as the dose at which any of the following appeared: severe nausea, vomiting, heartburn, headache, remarkable change in vital parameters such as blood pressure, heart rate, respiratory rate or oxygen saturation of blood, changes in QT interval, or any event that in the opinion of the investigators required stopping of the trial.

Maximum tolerated dose was defined as the dose before which any of the components of DLT appeared at a significantly higher rate as compared to placebo. In case this was not achieved, it was planned not to dose volunteers beyond 700 mg.

### 2.5. Study Procedure

The volunteers after giving informed consent were screened. The screening process was done latest within 1 month of admission for dosing. The volunteers who fulfilled the inclusion/exclusion criteria were admitted in the CPU the night before drug administration. Standardized dinner was served at 9 pm the night prior to dosing. On day 0, baseline blood sample for biochemical and haematological examination and stool and urine samples for routine and microscopy were taken. They received the study drug, according to the randomization sequence, under supervision. The empty packaging was returned to the container. The volunteers received drug as per randomization. Breakfast was given half an hour after drug administration. Subsequently, standardized lunch and dinner were served to the volunteers.

For pharmacokinetic (PK) study, eligible participants were admitted and housed in the clinical pharmacology unit an evening prior to the PK study. A standard dinner was served and a fasting condition for at least 10 hrs prior to dosing and 4 hrs after dosing was ensured. A peripheral forearm vein was cannulated for sampling for the PK analysis. 200 mg of the study drug was administered with 300 mL of water. The time of actual dose administration to the nearest minute was recorded. 2.5 mL of blood was collected from the indwelling cannula at 0 (before dose), 0.25, 0.5, 0.75, 1, 1.5, 2, 4, 6, 8, 12, 18, 24, 48, 72, 96, 120, and 144 hrs (after dose). 10 mL of additional blood was collected at 0 (before dose) and 24 hr (after dose) for laboratory parameters for safety assessment and PK control sample. A time window of ±2 minutes was permitted for collecting the post dose samples from the scheduled time for PK analysis.

Plasma was separated from blood sample intended for PK analysis, within 1 hour of blood sampling, by centrifugation at 2000 rpm for 10 min at 4°C. Samples were stored at −80°C pending transportation. Heparinised blood samples were transported to the laboratory within 7 days of collection.

### 2.6. Subject Monitoring

The subjects were attached to a cardiac monitor and vital HR, BP, ECG, oxygen saturation, and RR were recorded. Corrected QT interval using Fridericia's formula was calculated. Adverse events were assessed at specific time points (0.5, 1, 2, 4, 6, 8, 12, and 24 hrs after medication). Unscheduled monitoring was undertaken whenever it felt necessary. The volunteers were discharged after 24 hrs and they had to return at 48 hrs and on the 7th day for follow-up. At the end of the 7th day, they were said to have completed the study. Routine haematological and biochemical investigations were performed at 0 hr, 24 hrs after drug administration, and on day 7. The study was monitored by a Data Safety Monitoring Board.

### 2.7. Pharmacokinetic Analysis

Quantitative analyses of clinical phase ascending dose and single dose PKs samples were done at GLP lab of Pharmacokinetics and Metabolism Division, CDRI, Lucknow, India. *α*-Arteether was used as an internal standard and all the analyses were performed as per the published method by Singh et al. 2009 [19]. Acceptance criteria for analytical runs were fixed as per internationally approved guidelines [20]. Data acquisition and quantitation were performed using analyst software version 1.4 (Applied Biosystems, MDS SCIEX Toronto, Canada). PK parameters were estimated by using WinNonlin software (Version 1.5, Pharsight Corporation, Mountain View, CA). Oral data were best fitted in noncompartmental model. The individual plasma concentration-time profiles were subjected to noncompartmental analysis to determine various PK parameters. The PK parameters included the terminal elimination half-life (*T*_1/2_), maximum plasma concentration (*C*_max⁡_), time to reach maximum plasma concentration (*T*_max⁡_), area under the curve (AUC_0–*∞*_), and mean residence time (MRT).

### 2.8. Statistical Analysis

Continuous data were presented as mean ± SD and categorical data were presented as *n* (%). For comparison between the drug and placebo at each dose level, unpaired *t*-test was used for continuous variables and Fischer's exact test for categorical variables. For comparison between various dose levels, ANOVA was used. *P* value <0.05 was considered significant.

## 3. Results

**Figure 2:**
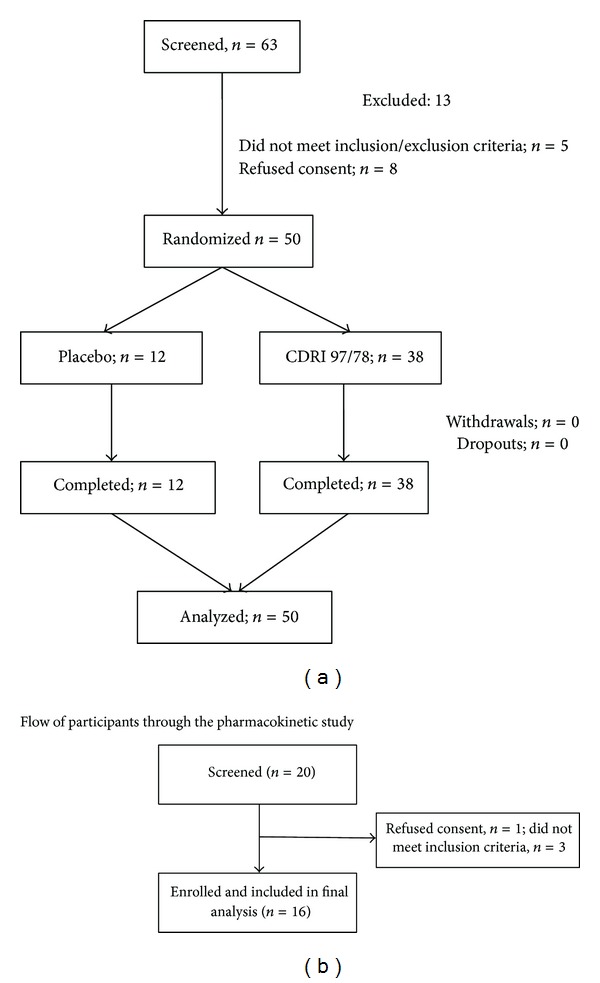
Volunteer screening flowchart: for ascending dose study, 50 normal healthy human volunteers were screened from 63 volunteers, while for single dose pharmacokinetics study 16 healthy volunteers were from 20 volunteers.

63 volunteers were screened out, of whom 50 volunteers matched the inclusion/exclusion criteria and were included in the safety study (Figure 2). For the PK study, 16 healthy male volunteers were included after screening of 20 volunteers. As required by the study protocol, all the volunteers were male (Figure 2). There was no significant difference in the demographic parameters like age (*P* = 0.7), height (*P* = 0.5), weight (*P* = 0.5), and body mass index (*P* = 0.3) among participants in various treatment groups (Table 1).

### 3.1. Adverse Events 

Adverse events that occurred during the study were few (Table 2) and largely of severity grade 1 to 2 (Table 2). There was no significant difference in the incidence of events in the drug and the placebo groups (*P* = 0.43).

Sedation was the most common adverse event reported across different groups (3/14; 21.4%). It was noted in 3 volunteers given 500 mg CDRI 97/78, 600 mg CDRI 97/78, and placebo about 2 hours after drug administration and lasted for 1-2 hours. It was grade 1-2 in severity, spontaneous and complete recovery was seen in all, and no action was required. The other adverse events noted were mild drowsiness, mild nausea, slight heaviness of head, bradycardia (56-57 bpm), visual disturbances, and itching (Table 3).

Severe adverse event, nausea and retching immediately after drug administration, was observed in one patient which lasted for 10 minutes; the patient also complained of dizziness and slight heaviness of head 1 hour after drug administration. On decoding, he was found to have received placebo. The symptoms resolved spontaneously; hence, no further action needed to be taken.

### 3.2. Clinical Parameters

At baseline, the different treatment groups were similar in terms of clinical parameters (pulse rate, blood pressure, and respiratory rate). None of the treatment groups demonstrated a significant alteration in pulse rate, respiratory rate, and systolic blood pressure at any time point compared to baseline. However, there was a significant change in diastolic blood pressure in 600 and 700 mg CDRI 97/78 group (76.87 ± 8.74, 78.37 ± 8.86, 79.25 ± 13.46, 67.12 ± 6.49, 73.12 ± 12.32, 75.87 ± 8.11, 74 ± 11, and 75 ± 10.03 mmHg at 0, 0.5, 1, 2, 4, 6, 8, and 12 hours, respectively; *P* = 0.02), although clinically significant decline was not observed in any of the volunteers. Statistical significance was observed for between group comparison of systolic and diastolic blood pressures 2 hours after drug administration. The SBP for 80 mg, 160 mg, 320 mg, 400 mg, 500 mg, 600 mg, 700 mg, and placebo groups were 112.17 ± 6.24, 119.5 ± 5.17, 127.5 ± 6.38, 111.83 ± 7.91, 114.5 ± 8.34, 112.83 ± 6.18, 115.5 ± 2.12, and 116.33 ± 8.77, respectively (*P* = 0.01). The DBP for 80 mg, 160 mg, 320 mg, 400 mg, 500 mg, 600 mg, 700 mg, and placebo groups were 73 ± 5.21, 77.17 ± 8.06, 82.33 ± 4.68, 71.33 ± 8.19, 71.83 ± 7.81, 66.5 ± 6.53, 69 ± 8.48, and 73.75 ± 8.24 (*P* = 0.03). Respiratory rate was found to be significantly different between treatment groups 4, 6, and 8 hours after drug administration, although this was not clinically relevant.

There was no statistically or clinically significant difference noted in the corrected QT interval within various treatment groups (*P* = 0.656; 0.523; 0.901; 0.348; 0.809; and 0.43, respectively, for 80, 160, 320, 400, 500, and 600/700 mg groups).

### 3.3. Hematological Parameters

Hemoglobin values demonstrated a statistically significant alteration during posttreatment period in the group receiving 400 mg CDRI 97/78 (14.25 ± 0.75, 14.92 ± 0.94, and 14.35 ± 1.10 gm% at baseline, 24 hr, and 7th day, respectively; *P* = 0.02). A statistically significant change in platelet counts was also observed in 320 mg CDRI 97/78 group (190 ± 68, 190 ± 69, and 212 ± 66 (×10^12^)/L, respectively; *P* = 0.04). All these values were well within normal limits. Other haematological parameters, which showed statistically significant difference, however, remained well within normal range, and did not demonstrate any dose response relationship, were PCV, total leukocyte and lymphocyte counts, RBC counts, and methemoglobin levels.

### 3.4. Biochemical Parameters

There was no significant difference in serum bilirubin, SGOT, and SGPT at any time point of measurement for intergroup comparisons. The values of serum alkaline phosphatase, however, were significantly different between various groups at all pre- and postdrug measurements. Post hoc analysis for this revealed that the results were significant for comparisons between lowest (80 mg CDRI 97/78) and higher doses (500 and 600 mg CDRI 97/78), although none of the groups was significantly different from placebo. All the values remained within normal range (Table 4). The other biochemical parameters which showed significant changes between groups were serum albumin, serum globulin, and blood glucose and serum HDL levels. However, they remained within the normal laboratory range and did not cause any sign or symptoms, and there was no dose response relationship noted.

### 3.5. Serum Electrolyte Levels

The groups demonstrated significant variation in serum sodium and serum potassium values at all time points of measurements. None of the volunteers had values outside the normal physiological ranges nor presented with any signs or symptoms of dyselectrolytemia, so these findings were considered clinically irrelevant.

### 3.6. Pharmacokinetic Analysis

**Figure 3:**
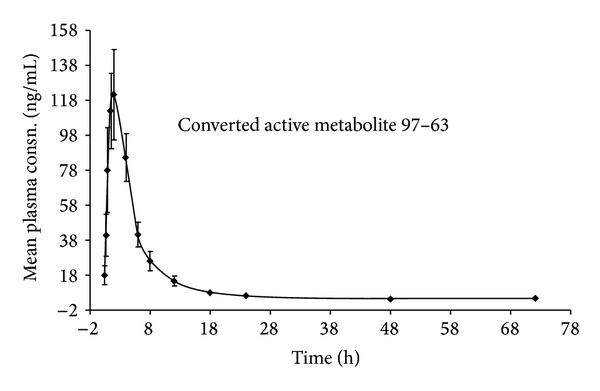
Mean plasma concentration-time profile of* in vivo* active metabolite 97/63 after oral administration of 97/78 at 200 mg single dose to male healthy human volunteers (*N* = 16).

Following oral administration at 200 mg, compound 97/78 was rapidly absorbed, gets immediately converted into active metabolite 97/63, and is eliminated from the systemic circulation within 48 h except in two volunteers (72 h), as observed from plasma concentration-time profile (Figure 3). PK parameters generated in individual volunteers have been shown in Table 5. *T*_max⁡_ of active metabolite 97/63 was found to be 2.28 ± 0.27 h with *C*_max⁡_ of 143.93 ± 28.32. Mean values of *T*_1/2_, MRT, and AUC_0–*∞*_ of 97/63 were 5.05 ± 0.57 h, 9.73 ± 1.06 h, and 845.22 ± 142.96 ng·h/mL, respectively. Active metabolite 97/63 was quantified from 0.5 h and up to 48 h in healthy volunteers with exception to two volunteers in whom it was observed up to 72 h. No additional metabolite peaks were observed during analysis except 97/63. In most of the volunteers, parent molecule 97/78 was below the limit of quantitation. Moreover, very low concentrations of 97/78 were observed in some volunteers, but these were not sufficient for generating the PK parameters.

## 4. Discussion

This was a first-in-human study conducted on healthy male volunteers for a new antimalarial drug, CDRI 97/78. Overall, the drug was found to be safe.

Few adverse events were reported which were of mild nature and were probable in causality. But the adverse events were neither clinically nor statistically significant. The 14 adverse events reported in this trial occurred in various treatment groups over a large time frame. Hence, it cannot be said definitely whether they were due to the drug or not. Moreover, similar adverse events were also seen in the placebo group. Even the statistically significant alteration in blood pressure was not associated with signs and symptoms of altered blood pressure and the values fell within normal ranges.

For none of the adverse events, any intervention in the form of drug administration, fluid substitution, and resuscitation was required. All the reported events continued to be observed and resolved spontaneously.

The severity of the adverse events was at best graded at 2. No serious adverse events were reported. The NCE under evaluation is a derivative of artemisinin compound which is rarely associated with any SAE. However, structural similarity is just an indicator for predicting adverse effect profile of a novel compound. A future possibility of such reactions coming into light always remains.

Hence, the MTD could not be determined. Though determination of MTD was the main purpose of the study, this could not be achieved. This is a definite possibility in phase I studies as has been seen previously [21, 22]. For the determination of dose for multiple ascending dose study, data from preclinical efficacy studies will be used, which is found to lie well within the highest dose evaluated in the present study.

There were some haematological and biochemical parameters which showed statistically significant values when compared to placebo but the differences were not clinically significant as most of them stayed within the normal laboratory range. Further, the absence of dose response relationship highlighted the fact that they were innocuous. Nonclinical toxicity data serves as a guide to the monitoring of adverse drug events once the studies are conducted in healthy volunteers. Though there were no specific concerns raised in nonclinical toxicity, a thorough evaluation of haematological and biochemical parameters was undertaken. Statistically significant differences should be interpreted with caution keeping in mind the clinical relevance [23, 24].

Since structurally the compound was similar to artemisinin, which has been reported to prolong QTc interval [25], a careful evaluation of QT interval was also undertaken. This was also undertaken for assessing a future need to undertake a thorough QT study. No clinically meaningful alteration of QT interval as laid down in E 14 guidance document for QT evaluation [26] was observed.

## 5. Conclusion

In conclusion, we can say that the new compound, CDRI 97/78, appears to be a safe and tolerable drug. Its safety can be further tested in multiple dose studies and, if found tolerable, can be a good therapeutic option for malaria, especially malaria resistant to available drugs.

## Figures and Tables

**Table 1:** Demographic characteristics of the volunteers included in the studies.

Characteristic	80 mg	160 mg	320 mg	400 mg	500 mg	600 mg	700 mg	Placebo
	*N* = 6	*N* = 6	*N* = 6	*N* = 6	*N* = 6	*N* = 6	*N* = 2	*N* = 12
Age in years; Mean (SD)	28.5 (10.93)	27.5 (4.32)	25.0 (7.12)	25.2 (2.13)	21.3 (3.53)	25.2 (7.67)	23.0 (1.41)	25.4 (5.75)
Height in cm; Mean (SD)	170.5 (5.33)	167.5 (5.8)	166.7 (6.44)	167.3 (4.08)	167.0 (3.57)	170.2 (5.77)	171.0 (1.41)	171.3 (4.89)
Weight in kg; Mean (SD)	58.6 (9.72)	69.0 (13.57)	56.6 (8.84)	61.6 (7.17)	59.6 (6.47)	61.3 (12.67)	63.5 (2.12)	60.7 (8.43)
BMI (kg/m^2^); Mean (SD)	20.1 (3.04)	24.8 (5.89)	20.3 (2.14)	22.0 (2.35)	21.4 (2.5)	21.7 (0.36)	20.7 (2.75)	21.4 (3.4)

**Table 2:** Adverse events noted at various drug dosages of CDRI 97/78.

Treatment group	Type of adverse events	No. of events
80 mg CDRI 97/78	Heaviness of head	1
160 mg CDRI 97/78	None	
320 mg CDRI 97/78	Visual disturbance	1
400 mg CDRI 97/78	Drowsiness	1
	Bradycardia	1
	Itching	1
500 mg CDRI 97/78	Sedation	1
	Nausea	1
600 and 700 mg CDRI 97/78	Sedation	1
Placebo	Sedation	1
	Drowsiness	1
	Nausea	1
	Retching	1
	Dizziness	1
	Heaviness of head	1

**Table 3:** Description of all adverse events observed in the study.

Name of AE	Treatment group	Treatment given	Time (hrs) of onset after drug administration	Duration of AE (min)	Spontaneous reversal (yes or no)	Reversal complete (yes or no)	Severity grade	Causality assessment
Sedation	600 mg	None	2	60	Yes	Yes	1-2	Possible
Sedation	500 mg	None	2	120	Yes	Yes	1-2	Possible
Sedation	Placebo	None	2	120	Yes	Yes	1-2	Not drug related
Drowsiness	400 mg	None	2	60	Yes	Yes	1	Possible
Drowsiness	Placebo	None	1.5	140	Yes	Yes	1	Not drug related
Nausea	500 mg	None	0.25	20	Yes	Yes	1	Possible
Nausea	Placebo	None	Immediate	10	Yes	Yes	3	Not drug related
Heaviness of head	80 mg	None	0.25	15	Yes	Yes	1	Possible
Heaviness of head	Placebo	None	1	10	Yes	Yes	1	Not drug related
Visual disturbances	320 mg	None	36	2-3	Yes	Yes	1	Remote
Bradycardia	400 mg	None	1	60	Yes	Yes	1	Possible
Retching	Placebo	None	Immediate	10	Yes	Yes	3	Not drug related
Itching	400 mg	None	24	8	Yes	Yes	1	Possible
Dizziness	Placebo	None	1	10	Yes	Yes	3	Not drug related

Severity grading done according to Common Terminology Criteria for Adverse Events v4.0, US Department of Health and Human Services. Grade 1: Mild; asymptomatic or mild symptoms; clinical or diagnostic observations only; intervention not indicated. Grade 2: Moderate; minimal, local or noninvasive intervention indicated; Grade 3: Severe or medically significant but not immediately life-threatening; hospitalization or prolongation of hospitalization indicated; disabling. Grade 4: Life-threatening consequences; urgent intervention indicated. Grade 5: Death related to AE.

Causality assessment: None; remote (<5% chance of being drug related); possible (>5% but ≤50% chance of being drug related; probable (>50% chance of being drug related) and definite.

Grade 3 severity—volunteer was on placebo.

**Table 4:** Comparison of liver function tests between various treatment groups on repeated observations at baseline, 24 hours and day 7.

Treatment groups	Total serum bilirubin			Serum SGOT			Serum SGPT			Serum Alkaline phosphatase		
	Baseline	24 hour	Day 7	Baseline	24 hour	Day 7	Baseline	24 hour	Day 7	Baseline	24 hour	Day 7
80 mg (*n* = 6)	0.82 ± 0.24	0.73 ± 0.24	0.64 ± 0.23	22.15 ± 6.3	23.48 ± 2.88	27.7 ± 6.1	21.88 ± 10.0	26.2 ± 6.6	21.8 ± 3.7	95.5 ± 26.65	102.5 ± 30.09	106.83 ± 37.12
160 mg (*n* = 6)	0.76 ± 0.39	0.55 ± 0.07	0.6 ± 0.06	34.18 ± 7.89	33.98 ± 9.69	38.33 ± 8.91	37.21 ± 4.16	39.43 ± 8.08	44.5 ± 11.64	211.5 ± 68.65	212 ± 67	207.17 ± 61.03
320 mg (*n* = 6)	0.73 ± 0.52	0.8 ± 0.4	0.87 ± 0.59	32.83 ± 5.15	34.17 ± 7.76	35 ± 8.53	33.83 ± 9.09	37.33 ± 8.87	38.33 ± 13.84	242 ± 45.24	222.5 ± 34.55	235.83 ± 32.49
400 mg (*n* = 6)	0.52 ± 0.04	0.53 ± 0.05	0.55 ± 0.08	26.67 ± 6.62	31.5 ± 4.5	32.33 ± 8.87	29 ± 5.44	35.17 ± 4.12	38 ± 12.95	232.83 ± 42	251.67 ± 47.59	242.83 ± 34.82
500 mg (*n* = 6)	0.52 ± 0.04	0.52 ± 0.04	0.53 ± 0.05	31.33 ± 8.76	31.5 ± 6.02	34 ± 6.00	32.83 ± 11.5	34.83 ± 11.07	35.33 ± 10.56	321.17 ± 151	313 ± 124.5	339.67 ± 184.25
600 mg (*n* = 6)	0.8 ± 0.54	0.8 ± 0.59	0.82 ± 0.59	31.17 ± 6.3	30.33 ± 6.74	31.33 ± 5.78	30 ± 7.16	30.5 ± 8.46	33.17 ± 10.53	272.5 ± 66.71	275.83 ± 61.29	274 ± 75.17
700 mg (*n* = 2)	0.6 ± 0.14	0.6 ± 0.14	0.55 ± 0.07	35.5 ± 0.71	40 ± 4.24	34.5 ± 0.71	46 ± 5.66	46.5 ± 4.95	40 ± 11.31	261.5 ± 50.2	254 ± 56.57	260.5 ± 44.55
Placebo (*n* = 12)	0.55 ± 0.07	0.56 ± 0.06	0.55 ± 0.12	31.91 ± 9.67	31.17 ± 9.18	30.5 ± 8.15	37.08 ± 18.11	38.58 ± 19.56	35.83 ± 18.11	208 ± 68.23	208 ± 65.56	208.58 ± 72.23

ANOVA												
*F*	1.114	1.308	1.105	1.701	1.631	1.14	1.62	1.097	1.435	4.545	5.103	3.802
df	49	49	49	49	49	49	49	49	49	49	49	49
*P*	0.37	0.27	0.38	0.13	0.15	0.36	0.16	0.38	0.22	<0.001*	<0.001**	0.002^#^

*P = 0.002 for 500 mg compared to 80 mg drug and *P* = 0.02 for 600 mg compared to 80 mg drug (Scheffe's test); *P = 0.001 and 0.006 for 80 mg drug compared to 500 and 600 mg drug respectively (Scheffe's test). ^#^ *P* = 0.006 for comparison between 80 and 500 mg drug groups (Scheffe's test). No difference from placebo in any comparison.

**Table 5:** Pharmacokinetic parameters of converted active metabolite 97/63 after oral administration of 97/78 at 200 mg single dose to male healthy human volunteers (*N* = 16).

PK parameter	Converted *in vivo* metabolite, 97/63																Mean ± SEM
	Male healthy human volunteers (*N* = 16)															
	1	2	3	4	5	6	7	8	9	10	11	12	13	14	15	16
*C*_max⁡_ (ng/mL)	172	20.1	373	187	149	355	42.6	202	60.9	295	47.4	50.4	65.4	105	55	123	143.93 ± 28.32
*T*_max⁡_ (h)	1.5	4	1	2	1.5	2	4	4	4	2	2	2	1.5	1.5	2	1.5	2.28 ± 0.27
*T*_1/2_ (h)	13.89	2.17	4.78	2.20	3.88	21.27	3.00	8.94	7.80	11.63	24.26	14.50	10.10	17.73	24.51	18.90	11.85 ± 1.94
AUC_0−*∞*_ (ng·h/mL)	788.7	104.8	1644	602.6	785	1522.6	207.9	1604.9	511.7	1898.9	935	356.1	583.6	696.8	833.1	984.1	878.74 ± 133.15
MRT (h)	12.57	5.72	13.7	3.53	5.38	14.25	6.42	14.13	9.28	9.76	30.72	14.53	11.26	18.37	32.20	18.52	13.77 ± 2.05
